# Syndrome de Maffucci: des enchondromes à surveiller de près

**DOI:** 10.11604/pamj.2015.20.358.4813

**Published:** 2015-04-14

**Authors:** Mohamed Azouz, Moradh Elyaacoubi

**Affiliations:** 1 Service de Chirurgie Orthopédique et de Traumatologie, CHU Ibn Sina, Rabat, Maroc

**Keywords:** Syndrome de Maffucci, enchondromes, hémangiomatose, Maffucci syndrome, enchondroma, hemangiomatosis

## Image en medicine

Le syndrome de Maffucci est une affection très rare caractérisée par l'association de chondromes multiples et d'hémangiomatose des parties molles. Les os les plus atteints dans cette affection sont par ordre de fréquence décroissant: les os de la main, les os des pieds, le fémur, les os de la jambe, le bassin, l'humérus et les os de l'avant-bras. Le risque majeur du syndrome de Maffucci reste la transformation chondrosarcomateuse. Le traitement du syndrome de Maffucci reste limité aux antalgiques et aux interventions chirurgicales de résection tumorale et de correction des déformations. Nous rapportons le cas d'un patient de 21 ans, ayant consulté pour des déformations des deux mains évoluant depuis l'enfance. L'examen clinique a mis en évidence des déformations avec des tuméfactions des doigts associés à des hémangiomes cutanés au niveau du thorax et du bras droit. Les radiographies standard ont montré des multiples lacunes anarchiques soufflant les corticales et intéressant toutes les phalanges et les métacarpiens. La biopsie a permis de poser le diagnostic de chondrome et l'association de chondromes multiples et d'angiomes cutanés a fait évoquer le diagnostic de syndrome de Maffucci. Le traitement avait consisté en la résection chirurgicale des chondromes les plus saillants des deux mains. L’évolution était marquée par la récidive motivant une reprise chirurgicale. Le syndrome de Maffucci est une entité très rare, caractérisée par l'association d'une enchondromatose multiple et d'une hémangiomatose. Nécessitant une surveillance de prés en raison du risque de dégénérescence sarcomateuse aussi bien des lésions osseuses que cutanées.

**Figure 1:**
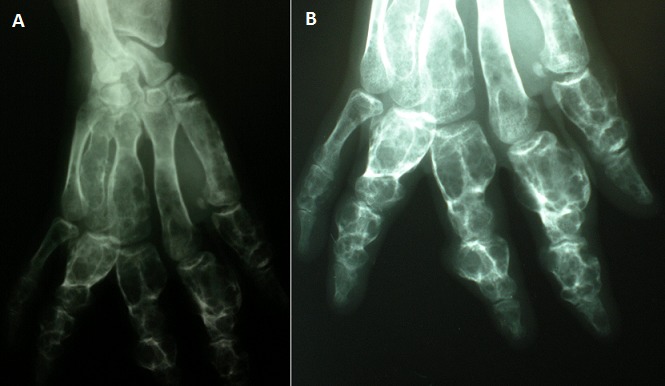
Syndrome de Maffucci: des enchondromes à surveiller de près

